# Anomalous Kidney

**Published:** 1906-04

**Authors:** Frank K. Boland

**Affiliations:** Atlanta, Ga.


### ANOMALOUS KIDNEY.

BY FRANK K. BOLAND, M.D.,
ATLANTA, GA.

The right kidney of a male negro subject, recently dissected at the Atlanta School of Medicine, was found lying obliquely across the bifurcation of the aorta, the hilum being directed slightly upward. It was normal in size, and presented a constriction on its right side to correspond with the hilum on its left, giving it the shape of the figure eight. An accessory ureteral duct began at the pelvis on the left side of the kidney, crossed over its front, and emerged from the depression on the right. The two ducts, the normal one and the accessory one, united a short distance below the kidney and took the usual course to the bladder. The right renal artery was given off from the aorta at the usual place, and coursed downward to reach the hilum at the kidney, which was situated just in front of the aorta. Similarly, the renal vein, after leaving the hilum, ran upward to unite with the vena cava at the usual place. The kidney was firmly fixed in its position, and in a healthy condition. The left kidney was normal.

---

It is a peculiar fact that post-operative prolapse through the epigastric wound occurs frequently in operations for malignant disease of the stomach. Such wound therefore should be closed with more than usual firmness and all possible precautions should be taken to guard against post-operative vomiting.—*American Journal of Surgery.*

